# The safety of Homnawakod herbal formula containing *Aristolochia tagala* Cham. in Wistar rats

**DOI:** 10.1186/1472-6882-12-170

**Published:** 2012-10-03

**Authors:** Pinpat Tripatara, Winita Onlamul, Suksalin Booranasubkajorn, Jantanee Wattanarangsan, Sukit Huabprasert, Natchagorn Lumlerdkij, Pravit Akarasereenont, Tawee Laohapand

**Affiliations:** 1 Department of Pharmacology, Faculty of Medicine Siriraj Hospital, Mahidol University, Bangkok, Thailand; 2 Center of Applied Thai Traditional Medicine, Faculty of Medicine Siriraj Hospital, Mahidol University, Bangkok, Thailand

**Keywords:** Aristolochia, Homnawakod, Aristolochic acid, Nephrotoxicity, Dose, HPLC, LC/MS

## Abstract

**Background:**

A dried root of *Aristolochia tagala* Cham. (ATC) is often used in Thai traditional medicine as an antipyretic, anti-inflammatory agent, muscle relaxant, appetite-enhancing agent, and analeptic. Homnawakod, an important herbal recipe, originally contains ATC in its formula, however, some *Aristolochia* species have been reported to cause nephrotoxicity due to aristolochic acid (AA) and its derivatives, resulting in ATC removal from all formulae. Therefore, this study investigates the chemical profiles of ATC, the original (HNK+ATC) and the present Homnawakod Ayurved Siriraj Herbal Formulary™ (HNK), and investigates whether they could cause nephrotoxicity or aggravate LPS-induced organ injuries *in vivo*.

**Methods:**

HPLC and LC/MS were used for chemical profile study. Male Wistar rats were randomly divided into groups in which the rats were intragastrically administered distilled water (2 groups), ATC (10 or 30 mg/kg), HNK+ATC (540 or 1,620 mg/kg), or HNK (1,590 mg/kg) for 21 days. A positive control group was administered with single dose 100 mg/kg standard AA-I intragastrically at day 1. Serum creatinine and urea were measured at baseline and at 7, 14 and 21 days of the treatment. On day 22, a model of lipopolysaccharide (LPS)-induced endotoxemia was used. One-way and two-way analyses of variance were performed and a *P* value of less than 0.05 was considered to be significant.

**Results:**

The similarity of the HPLC chromatograms of HNK+ATC and HNK could suggest that the qualities of both formulae are nearly the same in terms of chemical profile. The amount of AA-I found in ATC is 0.24%w/w. All experimental groups exhibited similar levels of serum urea at baseline and 7 and 14 days of the treatment. At 21 days, rats received AA exhibited a significant increase in serum urea, whereas the others did not exhibit such toxicity. On day 22, there were no significant changes in LPS-induced renal and liver dysfunction, or LPS-induced mean arterial pressure (MAP) reduction upon administration of ATC, HNK+ATC, HNK or AA-I.

**Conclusions:**

These results suggest that ATC, HNK+ATC or HNK, at the animal dose equivalent to that used in human, do not cause the acute nephrotoxicity in rats and do not aggravate LPS-induced organ injuries even further.

## Background

Krai-krue is a dried root of *Aristolochia pierrei* Lecomte or *Aristolochia tagala* Cham. (ATC) in the family Aristolochiaceae [1]. Krai-krue is one of the crude drugs often used in Thai traditional medicine. It is used as an antipyretic, anti-inflammatory agent, muscle relaxant, appetite-enhancing agent, and analeptic [2]. Many important Thai traditional medicine recipes contain krai-krue in their formulae such as: original Homnawakod, Homintrajak, Thartbanchob, and Ammaruekkhawatee.

Like other *Aristolochia* species [3], ATC contains aristolochic acid (AA) and derivatives [2]. Unfortunately, this AA and its derivatives have been reported to contribute to the nephrotoxicity and carcinogenicity of some *Aristolochia* species [4-7]. Aristolochic acid nephropathy is a rapidly progressive interstitial nephropathy. It was reported for the first time in 1992 as “Chinese herb nephropathy” because it involved patients who consumed Chinese herb products containing *Aristolochia* species. This caused a controversy in using herbal recipes containing ATC in traditional medicine.

Homnawakod is a famous Thai traditional herbal remedy used in vertigo, nausea and vomiting treatment. It has been widely used in Thailand for a long time, with no report of undesirable symptoms [8]. The formula has been included in the List of Herbal Medicinal Products since 2006 by National Drug Committee of Thailand. Homnawakod Ayurved Siriraj Herbal Formulary™ is a mixture of sodium borate anhydrous and 55 herbs (Table 1) available in powder and tablet dosage forms. It should be used with caution in pregnant women and pollen-hypersensitive patients. In 2011, the National Drug Committee removed krai-krue from every Thai traditional herbal recipe. Thus, Ayurved Siriraj™ has removed ATC from its formulae since that time.

**Table 1:** **List of ingredients of Homnawakod Ayurved Siriraj Herbal Formulary™**

		
*Alyxia reinwardtii* Blume	*Euphorbia antiquorum* L.	*Pimpinella anisum* L.
*Amomum krervanh* Pierre	*Foeniculum vulgare*	*Pinus spp*.
*Anacyclus pyrehtrum* (L.) Lagasca	*Glycyrrhiza glabra* L.	*Piper chaba* Hunt
*Anethum graveolens* L.	*Gymnopetalum chinense*	*Piper ribesoides* Wall., *Dalbergia.*
*Angelica dahurica* Benth.	*Jasminum sambac* (L.)	*Piper sarmentosum* Roxb.
*Angelica sinensis* (Oliv.) Diels	*Kaempferia galanga* L.	*Plantago ovata* Forssk.
*Aquilaria crassna*	*Lepidium sativum* L.	*Plumbago indica* L.
*Aristolochia tagala* Cham.	*Ligusticum sinense*	*Saussurea lappa* C.B.Clarke
*Artemisia annua* L.	*Maclura cochinchinensis* (Lour.) Corner	*Sophora tomentosa* L.
*Atractylodes lancea*	*Mammea siamensis* Kostern.	*Syzygium aromaticum* (L.)
*Brucea javanica* (L.) Merr.	*Mesua ferrea* L.	*Terminalia bellirica* (Gaertn.) Roxb.
*Carum carvi* L.	*Mimusops elengi* L. (flower and wood)	*Terminalia chebula* Retz.
*Cinnamomum bejolghota*	*Mollugo pentaphylla* L.	*Tinospora crispa* (L.)
*Cinnamomun loureirii* Nees	*Myristica fragrans* Houtt. (mace, nutmeg and wood)	*Trachyspermum ammi* (L.) Sprague
*Coriandrum sativum* L.	*Nelumbo nucifera* Gaertn.	*Vetiveria zizanioides* (L.) Nash ex Small
*Cuminum cyminum* L.	*Nigella sativa* L.	*Zingiber officinale* Roxb.
*Cyperus rotundus* L.	*Phyllanthus emblica* L.	
*Dracaena loureiri* Gagnep.	*Picrorhiza kurrooa* Royle ex Benth.	

According to Thai traditional medicine theory that the taste of drug is correlated with the pharmacological action [9], ATC, which has a bitter, pungent and cool taste, would act on blood and bile, and act as antipyretic and appetite stimulator. In original Homnawakod, ATC is an auxiliary drug which is a component that supports the main ingredients [9], so removing ATC from the formula may affect the balance of the recipe. Thus quality, safety and efficacy assessments of the new formula from which ATC was removed are needed.

The objectives of this study are i) to assess the quality of the recipes by investigating the differences of chemical pattern and the presence of AAs in ATC, the original Homnawakod Ayurved Siriraj Herbal Formulary™ (HNK+ATC) that contains ATC, and the present Homnawakod Ayurved Siriraj Herbal Formulary™ (HNK), and ii) to assess the safety of the formulae by investigating whether ATC, HNK+ATC or HNK causes nephrotoxicity in rats. This is of importance in adding to the scientific knowledge about the use of the Homnawakod formula, and providing information that can be applied to other studies of ATC-removed traditional herbal formulae in the future.

## Methods

### Test substances and chemicals

ATC, HNK+ATC, and HNK were prepared by Ayurved Siriraj™ Manufacturing Unit of Herbal Medicine and Products, Center of Applied Thai Traditional Medicine, Faculty of Medicine Siriraj Hospital, Mahidol University, Bangkok, Thailand (GMP certified since 2009).

All plant materials were purchased from Tai-hua-jan drugstore and authenticated by two experienced Thai traditional practitioners using macroscopic identification and organoleptic techniques which based on anatomical characteristics of the individual plant parts and color, fracture, smell, or taste. Then the specimens were sorted, washed and oven-dried. The cleaned dried roots of ATC were ground into powder. For HNK+ATC and HNK powder preparation, all components were accurately weighed according to the master formula and then ground together. The test specimens were kept in dry place, at room temperature and protected from light until use.

Reference substances, aristolochic acid I (A5512), aristolochic acid I sodium salt (A9451) and aristolochic acid mixture of I and II (R751669) were purchased from Sigma (St. Louis, USA). Other reagents were methanol (HPLC grade, Scharlau, Spain), acetic acid (analytical grade, Merck, Germany) and Milli-Q water (from Milli-Q water system, Millipore, France).

### LC/ MS analysis

ATC, HNK+ATC and HNK powders were extracted three times with methanol, under sonication (30 minutes per one extraction). After being centrifuged at 4,000 rpm for 10 minutes at 4°C, supernatants were filtered through the 0.2 μm polyvinylidenedifluoride filters. The final concentrations of the sample solutions were 1.83 mg/ml for ATC, 100 mg/ml for HNK+ATC and 100 mg/ml for HNK. Liquid chromatography (LC) analyses were carried out using an ultra high performance liquid chromatography (UPLC) system (Acquity UPLC®, Waters, Milford, MA, USA) equipped with a binary solvent delivery system, an auto sampler and a photodiode array detector (Acquity UPLC^®^). The separations were performed on a reverse phase column (Acquity UPLC^®^ HSS T3, 1.8μm, 2.1 x 50 mm, Waters, Ireland). The mass spectrometry (MS) analyses were carried out on a single quadrupole detector (SQD) mass spectrometer (Waters) equipped with a Z-spray electrospray interface (ESI). The analyses were done in positive mode, and the data were acquired by single ion recording (SIR). The ionization source working conditions were as following: capillary voltage, 3 kV; source temperature, 130°C; cone gas flow rate, 10 L/h; desolvation gas flow rate, 600 L/h; and desolvation temperature, 350°C. Nitrogen gas (>99.9% purity) was used. Cone voltage was optimized by infusion of 1 μg/mL standard AA-I solution in methanol. The mobile phase was 0.1% acetic acid in Milli-Q water and methanol with a gradient program of 5 – 100% methanol in 20 minutes. The flow rate was 0.25 mL/min. The method was modified from Koh HL., et al., 2006 [10].

### Animal care

Ninety-four male Wistar rats (180–250 g) were used in this study. They were purchased from the National Laboratory Animal Center, Nakorn Pathom, Thailand, were used in this study. All animals received a standard diet and water *ad libitum*, and were cared for in accordance with *the UK Home Office Guidance in the Operation of the Animals (Scientific Procedures) Act 1986*. Rats were housed in the animal care facility at the Department of Anatomy, Faculty of Siriraj Medicine Hospital, Mahidol University under standard conditions*.* The animals were acclimatized at least one week before the experiments. The study protocol was reviewed and approved by Siriraj Animal Care and Use Committee.

### Animal preparation and treatments

After one week acclimatization, the rats were randomly divided into seven groups and received daily intragastric treatment of different compounds for 21 days and received lipopolysaccharide (LPS) or vehicle on day 22 as following;

● **ATC(10)+LPS**: Rats received ATC (10 mg/kg/day) for 21 days and LPS (6 mg/mL/kg) on day 22.

● **ATC(30)+LPS**: Rats received ATC (30 mg/kg/day) for 21 days and LPS (6 mg/mL/kg) on day 22.

● **HNK+ATC(540)+LPS**: Rats received HNK+ATC (540 mg/kg/day containing 10 mg/kg/day ATC) for 21 days and LPS (6 mg/mL/kg) on day 22.

● **HNK+ATC(1620)+LPS**: Rats received HNK+ATC (1,620 mg/kg/day containing 30 mg/kg/day ATC) for 21 days and LPS (6 mg/mL/kg) on day 22.

● **HNK(1590)+LPS**: Rats received HNK (1,590 mg/kg/day) for 21 days and LPS (6 mg/mL/kg) on day 22.

● **Sham+LPS**: Rats received sterile water for 21 days and LPS (6 mg/mL/kg) on day 22.

● **Sham+vehicle**: Rats received sterile water for 21 days and vehicle of LPS (saline) on day 22.

The lower dose (10 mg/kg) of ATC was equivalent to the amount of ATC in the highest dose of HNK+ATC which was used in humans. The dose translation from human to animal dose was based on body surface area according to the formula as demonstrated in the literature [11].

Another group for positive control was administered with single dose 100 mg/kg standard AA-I intragastrically at day 1 and LPS (6 mg/mL/kg) on day 22. The single dose of 100 mg/kg of standard AA-I is the dose shown to increase serum creatinine and urea as demonstrated [3].

The model of lipopolysaccharide (LPS)-induced endotoxemia and organ injuries [12] was modified to study the effects of AA-I, ATC, HNK+ATC and HNK on LPS-induced organ injuries. Briefly, on day 22 all groups of rats were anesthetized and treated intravenously with 6 mg/kg of LPS or saline. Mean arterial blood pressure (MAP) was measured throughout the experiment by a data acquisition system (PowerLab 8/30, Chart v6, AD Instruments, Bangkok, Thailand).

### Measurement of biochemical parameters

Blood samples were collected from tail vein for measurement of serum creatinine and urea at baseline and 7, 14, and 21 days of the treatment. On day 22, blood samples were collected via carotid artery catheter at 6 hours after LPS treatment for measurement of serum creatinine, urea, and alanine transaminase (ALT). The samples were centrifuged to separate the plasma. All plasma samples were frozen and stored at −80°C until analyzed for biochemical parameters by the standard laboratory in the Department of Clinical Pathology, Faculty of Medicine Siriraj Hospital.

### Data analysis and statistical methods

All values described in the text and figures are expressed as mean±SEM for N observations. Two-way analysis of variance with Bonferroni post *hoc-*test was performed on MAP data, and one-way analysis of variance with Dunnett's post *hoc-*test was performed on all other data using GraphPad Prism version 5.02 for Windows (GraphPad Software, http://www.graphpad.com); a *P* value of less than 0.05 was considered to be significant.

## Results

### LC/ MS analysis

**Figure 1:**
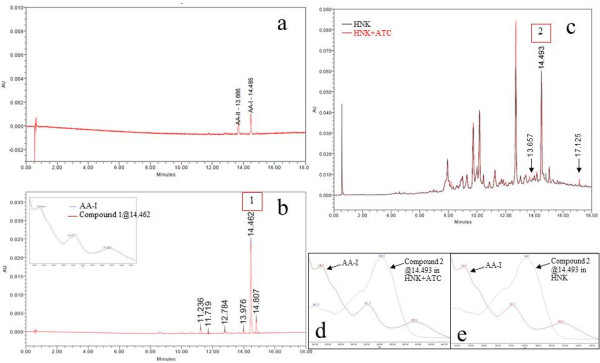
**HPLC chromatograms and UV spectra of AA standard and herbal samples.** HPLC chromatograms of (**a**) standard mixture of AA-I and AA-II (**b**) ATC and UV spectra (**c**) overlaid HNK+ATC and HNK (**d**) overlaid UV spectra of the peak at RT 14.510 in HNK+ATC and AA-I and (**e**) overlaid UV spectra of the peak at RT 14.510 in HNK and AA-I.

**Figure 2:**
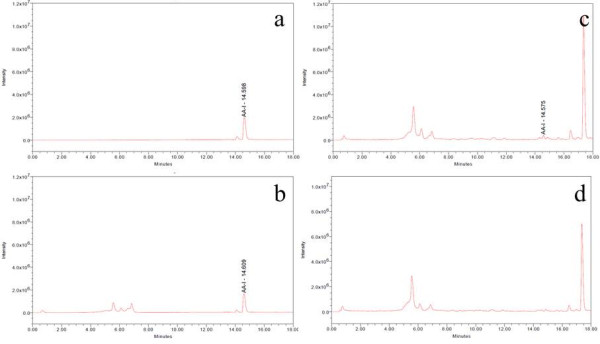
**Total ion chromatograms of AA-I standard and herbal samples at m/z 342.** Total ion chromatograms at m/z 342 of (**a**) AA-I (**b**) ATC (**c**) HNK+ATC and (**d**) HNK.

**Figure 3:**
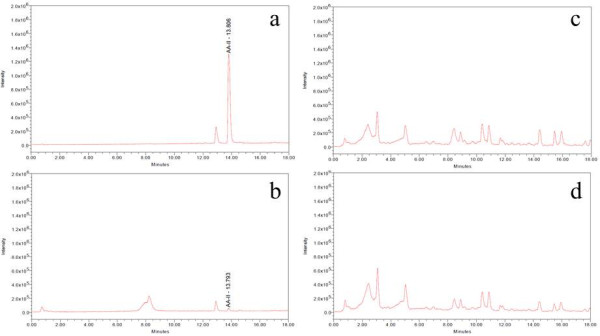
**Total ion chromatograms of AA-II standard and herbal samples at m/z 312.** Total ion chromatograms at m/z 312 of (**a**) AA-II (**b**) ATC (**c**) HNK+ATC and (**d**) HNK.

HPLC chromatographic fingerprint of standard mixture of AA-I and AA-II is shown in Figure 1a. The retention times (RT) of AA-I and AA-II are 14.485 and 13.666 respectively. Compounds 1 and 2 presented in the samples were identified by comparison of their RT and ultraviolet (UV) spectra with those of the authentic standard. The result shows that compound 1 in ATC (Figure 1b) was AA-I because its peak was at RT 14.462 which was close to that of AA-I and its UV spectrum was the same as AA-I spectrum (Figure 1b). Although there were peaks at RT 14.493 in both HNK+ATC and HNK (Figure 1c) which were close to those of AA-I, their UV spectra were different from AA-I spectrum (Figure 1d, 1e), therefore the peaks in HNK+ATC and HNK were not AA-I. Interestingly, the HPLC fingerprints of the two formulae were nearly similar from which it can be seen that the height of small peaks at RT 13.657 and 17.125 found in HNK+ATC are slightly lower in HNK (Figure 1c). Identification of AA-I and AA-II in samples by MS analysis was also performed (Figure 2 and 3). From single ion recording at m/z 342, there were AA-I peaks found in ATC (Figure 2b) and HNK+ATC (Figure 2c), but not in HNK (Figure 2d). AA-II peak was found only in ATC (Figure 3b). The quantitation of AA-I in ATC was done by LC analysis. The calibration curve of AA-I done by six concentrations of standard AA-I (2–25 mg/ml) showed an accurate linearity (R^2^ = 0.999623). The amount of AA-I found in ATC sample was 0.24 %w/w.

### Effect of ATC, HNK+ATC, or HNK on renal function

**Figure 4:**
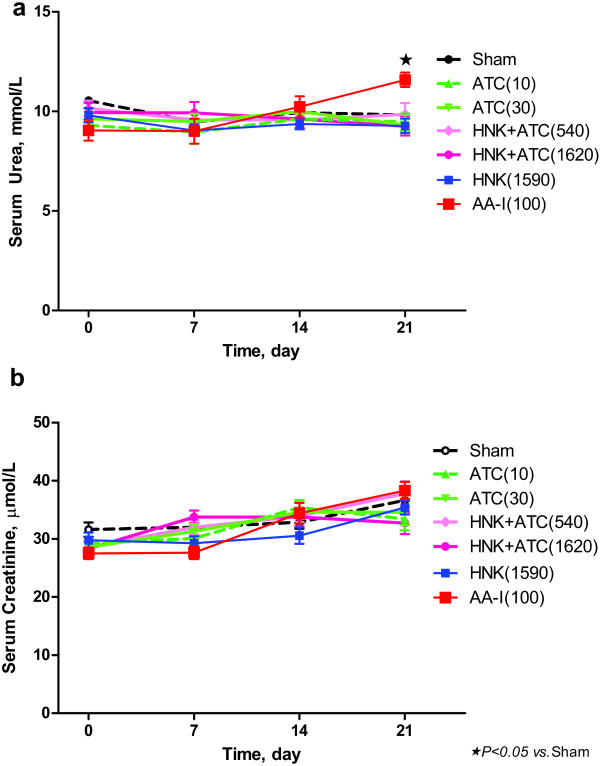
**Effect of ATC, HNK+ATC, or HNK on renal function.** Serum urea (**a**) and serum creatinine (**b**) were measured before and 7, 14 and 21 days after the treatment . Sham, *N* = 14; ATC(10), *N* = 10; ATC(30), *N* = 13; HNK+ATC(540), *N* = 13; HNK+ATC(1620), N = 11; HNK(1590), *N* = 13; and AA-I(100), *N =* 9).

There was no significant difference in serum urea (Figure 4a) or serum creatinine (Figure 4b) in groups treated with ATC, HNK+ATC, or HNK, when compared to vehicle-treated rats, either at baseline, or at the seventh, fourteenth, or twenty-first day of the treatment. At day 21 of the experiment, rats received AA-I exhibited a significant increase in serum urea but not serum creatinine, when compared to vehicle-treated rats.

### Effect of ATC, HNK+ATC, or HNK on renal dysfunction induced by LPS

**Figure 5:**
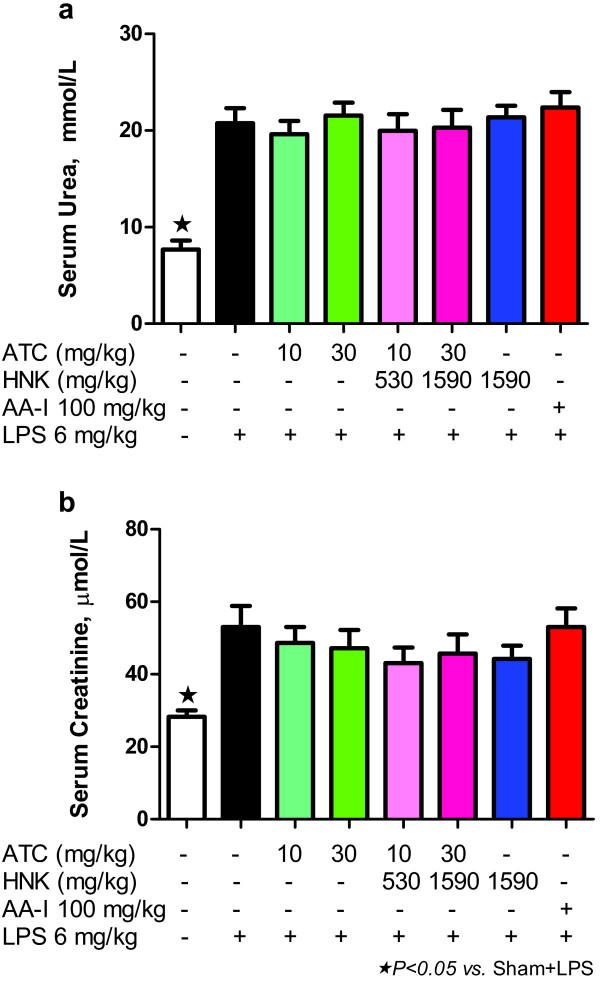
**Effect of ATC, HNK+ATC, or HNK on renal dysfunction induced by LPS.** Serum urea (**a**), and serum creatinine (**b**), were measured on day 22 of the experiment at 6 hours after LPS treatment. Sham+vehicle, *N* = 5; Sham+LPS, *N* = 9, ATC(10)+LPS, *N* = 8; ATC(30)+LPS, *N* = 11; HNK+ATC(540)+LPS, *N* = 9; HNK+ATC(1620)+LPS, *N* = 7; HNK(1590)+LPS, *N* = 11; and AA-I(100)+LPS, *N =* 9).

Rats that received LPS exhibited a significant increase in serum urea (Figure 5a), and serum creatinine (Figure 5b), indicating renal dysfunction. Treatment of rats with intragastric ATC, HNK+ATC, HNK or intravenous AA-I did not aggravate further injury caused by LPS.

### Effect of ATC, HNK+ATC, or HNK on abnormal liver function induced by LPS

**Figure 6:**
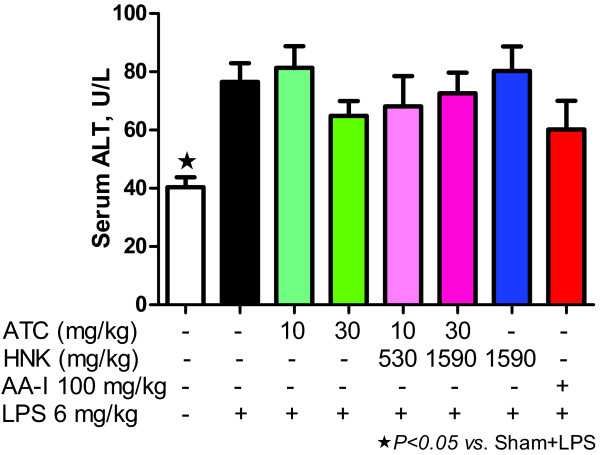
**Effect of ATC, HNK+ATC, or HNK on abnormal liver function induced by LPS.** Serum ALT was measured on day 22 of the experiment at 6 hours after LPS treatment. Sham+vehicle, *N* = 5; Sham+LPS, *N* = 9, ATC(10)+LPS, *N* = 8; ATC(30)+LPS, *N* = 11; HNK+ATC(540)+LPS, *N* = 9; HNK+ATC(1620)+LPS, *N* = 7; HNK(1590)+LPS, *N* = 11; and AA-I(100)+LPS, *N =* 9).

Rats that received LPS exhibited a significant increase in serum ALT (Figure 6), indicating liver injury. Treatment of rats with intragastric ATC, HNK+ATC, HNK or intravenous AA-I did not aggravate further injury caused by LPS.

### Effect of ATC, HNK+ATC, or HNK on the decrease in mean arterial blood pressure caused by LPS

**Figure 7:**
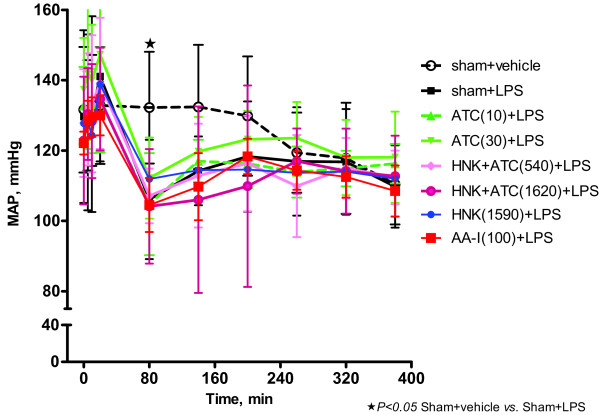
**Effect of ATC, HNK+ATC, or HNK on the decrease in MAP caused by LPS.** Mean arterial pressure was measured throughout the LPS experiment. Sham+vehicle, *N* = 5; Sham+LPS, *N* = 11, ATC(10)+LPS, *N* = 9; ATC(30)+LPS, *N* = 11; HNK+ATC(540)+LPS, *N* = 10; HNK+ATC(1620)+LPS, N = 8; HNK(1590), *N* = 11; and AA-I(100)+LPS, *N =* 8).

Overall, there was no significant difference in MAP between groups at baseline (Time = 0 min) (Figure 7). Intravenous administered LPS produced a significant decrease in MAP 1 h after the LPS administration (Time = 80 min) (Figure 5). Intragastric administration of ATC, HNK+ATC, HNK or intravenous AA-I did not cause further reduction in MAP (Figure 7).

## Discussion

Some *Aristolochia* species have been reported to cause nephrotoxicity and carcinogenicity due to AA and its derivatives [2,4-7]. A case of unexplained nephropathy was reported after two months ingestion of AA-containing *Aristolochia mollissemae* in a patient with long-standing Crohn's disease and recently diagnosed carcinoma of the colon [13]. This case contributed to the withdrawal of the AA-related herbs by the local health authority in Hong Kong [13]. Traditional medicine in Thailand has also used *Aristolochia* species in our herbal recipes. Specifically, in the current study, a dried root of ATC, namely Krai-krue, has been used in many herbal recipes such as Homnawakod, which has been included in the List of Herbal Medicinal Products since 2006 by the National Drug Committee of Thailand. In 2011, however, the National Drug Committee has removed krai-krue from every Thai traditional herbal recipe. Thus, Ayurved Siriraj™ has removed ATC from its formulae since last year, although it is still questionable whether the formulae really cause toxicity. More importantly, removing ATC from the formula may affect the balance of the recipe. Thus quality, safety and efficacy assessments of the new ATC-removed formula are needed.

For the quality assessment, HPLC chromatograms of ATC, HNK+ATC and HNK were analyzed. Although there was a peak in HNK+ATC and HNK at the RT close to the RT of AA-I, their UV spectra were different from AA-I spectrum. This finding reveals that the peak is not AA-I; however, it can be the result of the coelution of AA-I and another compound or more [14]. Taking into account this finding in which the interested peak was presented in both HNK+ATC and HNK, not in ATC, together with the finding that AA-I was clearly identified in ATC, it is most likely that the coelution occurs from the ingredients in HNK. The absence of AA-I and AA-II in the HPLC chromatograms of HNK+ATC and HNK indicates the limitation of LC/PDA for analyzing poly-herbal formula containing numerous ingredients. The MS may be a preferable technique. Nevertheless, there is a published method for analyzing traditional Chinese medicinal prescriptions that contain up to 17 ingredients with LC/PDA [15]. To prevent inconsistency in the ingredients quality, HNK+ATC and HNK were produced from the same batch of raw materials with the same procedure and the similarity of the chromatograms of both formulae suggests that their qualities are nearly the same. This indicates that the small portion of ATC, at only 1.83 %w/w, in HNK+ATC has only a slight effect on the overall chemical profile.

The MS analysis gave ion [M+H]^+^ at m/z 342 and m/z 312 for AA-I and AA-II, respectively. This is in agreement with the previous study [10,16]. LC/MS analysis shows that AA-I was presented clearly in ATC and HNK+ATC, while the peak of AA-II was found only in ATC but was too small to quantify. Therefore, the quantitation of AA-I was performed only in ATC and HNK+ATC. However, due to the complexity of the HNK+ATC, the amount of AA-I could be quantified only in the ATC sample as 0.24 %w/w, and the amount of AA-I in the formulae was obtained from calculation. The rats in the HNK(1590) group did not receive AA-I. The rats in the ATC(10) and HNK+ATC(540) groups received AA-I 0.024 mg/kg/day. Finally, the ATC(30) and HNK+ATC(1620) groups received AA-I 0.072 mg/kg/day.

Furthermore, we demonstrate here that HNK+ATC, HNK, or ATC itself does not cause nephrotoxicity in rats after daily intragastric administration for at least 21 days, whereas standard AA-I significantly increases serum urea at the twenty-first day of the experiment. A previous study was conducted with 4 g of dried stem of *Aristolochia manshuriensis* (which contained 4 mg of AA) administered daily for 5 days in 170 g female Wistar rats. The study showed the decrease in renal function within the first day after oral administration of the herb or 4 mg AA (23.53 mg/kg/day) [5]. In contrast, another study used 7 mg/kg/day of AA, subcutaneously injected once daily for 35 days. The serum creatinine levels at 10 and 35 days of the study were non-significantly increased compared with those of controls. The various study designs make the comparison among the studies difficult. The differences between our findings and the findings from previous literature may be attributed to the amount, route of administration and duration of AA-I that the rats received, together with the differences in the species of the herb and the herbal parts used. In the present study, the herb in the formulae is the dried root of *Aristolochia tagala* Cham. and, as stated above, the doses of AA-I were only 0.024 or 0.072 mg/kg/day. These amounts, which have already included a half-log scale higher dose, are much smaller than the reported toxic doses of AA-I in other literature, which range from 0.154 to 231 mg/kg in various study designs in rats [3]. This observation on the dose is the most notable reason why the formulae tested in the present study do not cause nephrotoxicity.

In addition, we demonstrate for the first time that ATC, HNK+ATC, HNK or AA-I did not aggravate intravenous LPS-induced increases of serum urea, creatinine, and ALT. The same observation also occurs for the reduction in MAP at 1 h after administration of LPS, which was not aggravated by ATC, HNK+ATC, HNK or AA-I. Although AA-I increased the level of serum urea at day 21 of the experiment, the injury was approximately 10% only. Here, LPS caused a two-fold increase of the level, and may mask the small effect of AA-I. However, it can be concluded that none of the treatment groups aggravate the renal and liver injuries and hypotensive effect caused by LPS.

Since the lower dose (10 mg/kg) of ATC used in this study was equivalent to the amount of ATC in the highest dose of HNK+ATC, which was used in humans, it is likely that the amount of AA-I human subjects received daily from this formula is conspicuously small. This is in accord with the fact that no adverse effect of this formula has been reported despite the fact that it has been used in Thai traditional medicine for a long time [8]. It should be noted that the duration of treatment in this study is 21 days which covers the duration of treatment (1–3 weeks) for most indications in human use. However, this study may not be applicable to the human exposed to the formula for longer than 21 days, such as in the use of this formula at a lower dose as a nutrient supplement. Nevertheless, *Aristolochia* species was reported to be nephrotoxic and was announced by the WHO to be carcinogenic in 2002 [9], so it was prohibited to be used in many countries worldwide. Therefore, AA-I analysis of any herbal formula is necessary in order to avoid the consumption of AA. Although removal of AA-containing herbs from the formulae is necessary, any modification should be carefully studied in order to avoid changes of efficacy. Our finding is an example of the study of the quality and safety of herbal formula modification.

## Conclusions

Herein, we demonstrate that both original and modified Homnawakod Ayurved Siriraj Herbal Formulary™, at the dose equivalent to that used in humans, do not cause acute nephrotoxicity or aggravate LPS-induced organ injuries in rats. Although the present Homnawakod Ayurved Siriraj Herbal Formulary™ seems to be safe, and should be used instead of the original one because it is free from AA-I, the efficacy study of the modified formula should be further investigated and compared to the original one in order to confirm that the modification does not change the efficacy of the formula.

## Abbreviations

AA: Aristolochic acid; A.D: *Anno Domini* (in the year of our Lord); ATC: *Aristolochia tagala* Cham; ALT: Alanine transaminase; β: Beta; °C: Degrees Celsius; ESI: Electrospray interface; G: Gram; GMP: Good manufacturing practice; H: Hour; HNK: Present Homnawakod Ayurved Siriraj Herbal Formulary™; HNK+ATC: Original Homnawakod Ayurved Siriraj Herbal Formulary™; HPLC: High performance liquid chromatography; IU: International units; Kg: Kilogram; L: Litre; LC: Liquid chromatography; LPS: Lipopolysaccharide; μ: Micro; m: Meter; MAP: Mean arterial blood pressure; Mg: Milligram; min: Minute; mL: Milliliter; mm: Millimetre; mmHg: Millimetres of mercury; MS: Mass spectrometry; m/z: Mass to charge ratio; N: Number of experiments or animals; %: Percentage; P: Probability; PMS: Phenylmethylsulfonyl fluoride; PVDF: Polyvinylidenedifluoride; R: Correlation coefficient; Rpm: Round per minute; RT: Retention time; SIR: Single ion recording; SEM: Standard error of mean; SQD: Single quadrupole detector; UPLC: Ultra high performance liquid chromatography; UV: Ultraviolet; w/w: Weight by weight.

## Competing interests

The authors declare that they have no competing interests.

## Authors' contributions

PT participated in the design of the study and coordination, interpretation of data, and drafted the manuscript. WO carried out the *in vivo* studies and performed the statistical analysis**.** SB and SH carried out the *in vivo* studies. JW carried out the quality studies. NL participated in the design of the study, interpretation of data, and helped to draft the manuscript. PV participated in the design of the study and helped to draft the manuscript. TL conceived of the study and provided the herbal samples. All authors read and approved the final manuscript.

## Pre-publication history

The pre-publication history for this paper can be accessed here:

http://www.biomedcentral.com/1472-6882/12/170/prepub
